# O Papel da Impressão 3D de Baixo Custo na Educação em Cardiopatias Congênitas

**DOI:** 10.36660/abc.20250805

**Published:** 2026-03-03

**Authors:** Vítor Ramos

**Affiliations:** 1 Leeds Teaching Hospitals NHS Trust - Paediatric Cardiology Reino Unido Leeds Teaching Hospitals NHS Trust - Paediatric Cardiology, Reino Unido

**Keywords:** Impressão Tridimensional, Cardiopatias Congênitas, Educação

A impressão tridimensional (3D) emergiu como uma tecnologia transformadora no tratamento de cardiopatias congênitas (CC), oferecendo novas possibilidades para visualização anatômica, planejamento de procedimentos e educação.^1–3^ Ao converter conjuntos de dados de imagem padrão em modelos físicos tangíveis, clínicos e residentes obtêm uma compreensão espacial intuitiva de malformações cardíacas complexas que muitas vezes são difíceis de interpretar usando apenas imagens bidimensionais.^4,5^

No entanto, a sustentabilidade de um programa de impressão 3D exige um planejamento financeiro cuidadoso. Os custos relacionados à infraestrutura, equipamentos, materiais e pessoal dedicado podem limitar a viabilidade a longo prazo.^6,7^ Felizmente, a crescente disponibilidade de softwares gratuitos e materiais de baixo custo reduziu significativamente as barreiras financeiras, tornando esses fluxos de trabalho mais acessíveis a instituições com recursos limitados.

Nesta edição, Cajueiro et al.^8^ apresentam um fluxo de trabalho inovador e de baixo custo para o desenvolvimento de modelos de CC impressos em 3D, com o objetivo de aprimorar o ensino. Sua abordagem utiliza software livre, impressoras 3D de mesa e materiais de impressão acessíveis, ampliando o acesso para pesquisadores e educadores em contextos com poucos recursos. Um painel de 26 médicos especialistas avaliou os protótipos iniciais por meio de um questionário estruturado, permitindo o refinamento sistemático dos modelos. As versões finais foram padronizadas e reunidas em kits educacionais otimizados para o ensino.^8^

Doze modelos de cardiopatia congênita foram desenvolvidos, incluindo defeito do septo atrioventricular e tetralogia de Fallot. Notavelmente, os autores empregaram uma técnica inovadora de mapeamento cartográfico de altura para converter imagens ecocardiográficas bidimensionais em mapas de volume tridimensionais e, ao fazer isso, abordaram parcialmente uma limitação conhecida da imagem seccional na representação precisa da anatomia valvar e subvalvar. A viabilidade desse fluxo de trabalho de baixo custo foi claramente demonstrada. Os residentes relataram melhor compreensão espacial e identificaram os modelos como ferramentas educacionais valiosas não apenas para residentes, mas também para familiares e estudantes de medicina. É importante ressaltar que os modelos estão disponíveis gratuitamente para download (http://sites.google.com/view/3dpequenoscoracoes), promovendo ampla disseminação de recursos educacionais em cardiologia pediátrica.

Apesar desses avanços, o termo "baixo custo" pode ser enganoso. O tempo continua sendo uma das barreiras mais significativas para a adoção rotineira. Intimamente ligado a isso está o requisito de conhecimento técnico avançado.^1,6,7^ A segmentação precisa da anatomia das CC exige conhecimento profundo tanto da morfologia cardíaca quanto de softwares sofisticados de processamento de imagens. Paradoxalmente, a impressão de baixo custo pode amplificar essa dependência, colocando ferramentas complexas em ambientes com suporte técnico limitado.^9^ Além disso, a ausência de currículos padronizados para a integração de modelos 3D no ensino de CC limita o impacto educacional consistente. Ironicamente, as regiões que mais se beneficiariam da impressão 3D acessível frequentemente carecem tanto de infraestrutura de imagem avançada quanto de pessoal treinado para manter tais programas.

A automação por meio de inteligência artificial (IA) poderia reduzir substancialmente o tempo e o trabalho de segmentação. No entanto, os modelos de IA atuais dependem de aprendizado profundo e permanecem severamente limitados em aplicações de CC devido à heterogeneidade intrínseca e à relativa raridade dessas condições. Como resultado, conjuntos de dados grandes e bem anotados, essenciais para um desempenho robusto da IA, são difíceis ou praticamente impossíveis de obter.

As tecnologias de realidade virtual e aumentada (RV/RA) podem oferecer uma solução complementar ou alternativa.^10–12^ Essas plataformas podem gerar modelos virtuais 3D diretamente a partir de dados DICOM brutos derivados de tomografia computadorizada (TC) e ressonância magnética (RM), evitando a segmentação trabalhosa exigida pela impressão 3D tradicional. Intervenções simuladas, como o projeto de patches cirúrgicos ou stents, já são possíveis nesses ambientes. Ironicamente, embora a TC e a RM sejam técnicas intrinsecamente tridimensionais, os visualizadores DICOM convencionais permanecem amplamente restritos à visualização bidimensional. Mesmo as reformatações multiplanares muitas vezes não conseguem transmitir a complexidade típica das CC. Novas plataformas de visualização agora permitem que os usuários salvem e revisitem múltiplos estados renderizados em volume 3D, aprimorando a interpretação longitudinal.^13^ À medida que a fronteira entre monitores tradicionais e headsets de RV/RA continua a se diluir, o acesso à modelagem 3D pode se expandir rapidamente em ambientes clínicos e educacionais com impactos comparativamente menores em custos e tempo.

Em conclusão, a impressão 3D de baixo custo, como um ganho futuro da IA e da visualização imersiva, apresenta uma promessa inegável para transformar o ensino e o tratamento de CC. No entanto, muitas vezes se subestimam os desafios relacionados ao tempo necessário, à dependência técnica, à integração no fluxo de trabalho e ao apoio institucional. Sem abordar essas barreiras fundamentais, a impressão 3D corre o risco de permanecer uma inovação poderosa, porém subutilizada, confinada a centros especializados em vez de incorporada à prática rotineira de CC. O progresso sustentável dependerá não apenas de impressoras mais baratas, mas também de fluxos de trabalho mais inteligentes, estratégias educacionais padronizadas e um compromisso institucional de longo prazo.
