# Supplementary material for: O Papel da Impressão 3D de Baixo Custo na Educação em Cardiopatias Congênitas
Source: Arq Bras Cardiol. 2026 Mar 3;123(1):e20250805. [Article in Portuguese] doi: 10.36660/abc.20250805 (PMC13127808; doi:10.36660/abc.20250805)
Supplement: Translation

# Translation: The Role of Low-Cost 3D Printing for Education in Congenital Heart Diseases

**DOI:** 10.36660/abc.20250805i

**Authors:** Vítor Ramos

**Affiliations:** 1 United Kingdom Leeds Teaching Hospitals NHS Trust - Paediatric Cardiology, United Kingdom

**Keywords:** Three-Dimensional Printing, Congenital Heart Defects, Education

Three-dimensional (3D) printing has emerged as a transformative technology in the management of congenital heart disease (CHD), offering new possibilities for anatomical visualization, procedural planning, and education.^1–3^ By converting standard imaging datasets into tangible physical models, clinicians and trainees gain intuitive spatial insight into complex cardiac malformations that are often difficult to interpret using two-dimensional images alone.^4,5^

However, sustaining a 3D printing program requires careful financial planning. Costs related to infrastructure, equipment, materials, and dedicated personnel can limit long-term feasibility.^6,7^ Encouragingly, the increasing availability of free software and low-cost materials has significantly reduced financial barriers, making these workflows more accessible to institutions with limited resources.

In this issue, Cajueiro et al.^8^ present an innovative low-cost workflow for developing 3D-printed CHD models aimed at enhancing education. Their approach relies on free software, desktop 3D printers, and affordable printing materials, broadening access for researchers and educators in low-resource settings. An expert panel of 26 physicians evaluated the initial prototypes using a structured questionnaire, allowing systematic refinement of the models. The final versions were standardized and assembled into educational kits optimized for teaching.^8^

Twelve CHD models were developed, including atrioventricular septal defect and Tetralogy of Fallot. Notably, the authors employed an inventive cartographic heightmap technique to convert two-dimensional echocardiographic images into three-dimensional volume maps and, by so doing, partially addressed a known limitation of cross-sectional imaging in accurately representing valvar and subvalvar anatomy. The feasibility of this low-cost workflow was clearly demonstrated. Residents reported improved spatial understanding and identified the models as valuable educational tools not only for trainees but also for families and medical students. Importantly, the models are freely available for download (http://sites.google.com/view/3dpequenoscoracoes), promoting broad dissemination of pediatric cardiology educational resources.

Despite these advances, the term "low-cost" can be misleading. Time remains one of the most significant barriers to routine adoption. Closely linked is the requirement for advanced technical expertise.^1,6,7^ Accurate segmentation of CHD anatomy demands deep knowledge of both cardiac morphology and sophisticated image-processing software. Paradoxically, low-cost printing may amplify this dependency by placing complex tools into environments with limited technical support.^9^ Furthermore, the absence of standardized curricula for integrating 3D models into CHD education limits consistent educational impact. Ironically, the regions that could benefit most from affordable 3D printing often lack both advanced imaging infrastructure and trained personnel to sustain such programs.

Automation through artificial intelligence (AI) could substantially reduce segmentation time and labor. However, current AI models rely on deep learning and remain severely hampered in CHD applications by the intrinsic heterogeneity and relative rarity of these conditions. As a result, large, well-annotated datasets, essential for robust AI performance, are difficult to virtually impossible to obtain.

Virtual and augmented reality (VR/AR) technologies may offer a complementary or alternative solution.^10–12^ These platforms can generate virtual 3D models directly from raw DICOM data derived from CT and MRI, avoiding the burdensome segmentation required for traditional 3D printing. Simulated interventions, such as designing surgical patches or stents, are already possible in these environments. Ironically, although CT and MRI are intrinsically three-dimensional techniques, conventional DICOM viewers remain largely restricted to two-dimensional visualization. Even multiplanar reformats often fall short in conveying the complexity typical of CHD. Newer visualization platforms now allow users to save and revisit multiple 3D volume-rendered states, enhancing longitudinal interpretation.^13^ As the boundary between traditional monitors and VR/AR headsets continues to blur, access to 3D modelling may expand rapidly across clinical and educational environments with comparatively lower impacts on cost and time.

In conclusion, low-cost 3D printing, as a future gain from AI and immersive visualization, holds undeniable promise for transforming CHD education and care. Yet it is often underestimated what are the realities of time consumption, technical dependency, workflow integration, and institutional support are. Without addressing these fundamental barriers, 3D printing risks remaining a powerful but underutilized innovation, confined to specialized centres rather than embedded in routine CHD practice. Sustainable progress will depend not only on cheaper printers, but on smarter workflows, standardized educational strategies, and long-term institutional commitment.
